# The Correlation between Serum Level of Leptin and Troponin in Children with Major Beta-Thalassemia 

**Published:** 2015-03-15

**Authors:** I Shahramian, NM Noori, A Teimouri, E Akhlaghi, E Sharafi

**Affiliations:** 1 Department of Pediatric, Zabol University of Medical Sciences, Zabol, Iran; 2 Department of Pediatric, Children and Adolescents Health Research Center, Zahedan University of Medical Sciences, Zahedan, Iran; 3 M.Phil, PhD in Demography, Children and Adolescents Health Research Center, Zahedan University of Medical Sciences, Zahedan, Iran; 4 General Physician, Faculty of Medicine, Zabol University of Medical Sciences, Zabol, Iran; 5 Resident of Ophthalmology, Zahedan University of Medical Sciences, Zahedan, Iran

**Keywords:** Leptin, Major beta thalassemia, Troponin

## Abstract

**Background:**

Polypeptide hormone Leptin suppresses inflammation in the heart muscle and protects heart from diseases. The purpose of this study is to evaluate the relationship between leptin and troponin serum levels with cardiac involvement in patients with major beta thalassemia.

**Materials and Methods:**

In this cross-sectional study, 70 children with major thalassemia were selected. Two ml blood was taken as sample from all children and after separating serum; the samples were maintained in -20°C temperature. Then, regarding cold chain conditions, the sample were sent to the Biochemistry Lab. Afterwards, leptin and troponin serum levels with the relevant kits and BMI were measured in all children, and information about age and gender was recorded. Collected data were analyzed with SPSS.

**Results:**

The mean of leptin in girls and boys were 2.47 ± 3.13 and 0.96 ± 1.08 respectively which showed a significant difference (t=2.74, p =0.009). A significant correlation was also observed between BMI and leptin (r = 0.374, P = 0.002). Another significant association was found between leptin and age (r = 0.248, P = 0.041). However, a significant inverse correlation between serum ferritin and age (r = - 0.607, P = 0.0001) was discovered. No correlation was found between leptin, troponin, and ferritin.

**Conclusion:**

Since Leptin increases with the advent of cardiac involvement and independent from troponin T, it can be a predictive marker of cardiac involvement in patients with major beta thalassemia.

## Introduction

Leukemia causes a significant decrease in the Major Beta - thalassemia is the most common hereditary hemoglobinopathies. About 100000 babies are born with this problem around the world annually (1-5). Leptin is a kind of protein with 146 amino acid and a multi- functional polypeptide hormone which regulates factors such as fat, energy homeostasis ,blood pressure, lipid and sugar metabolism, and bone mass and safety (1 ,3 ). This leptin hormone is produced by fat cells in various tissues (6). Moreover, epicard generates adipocytes in heart which plays an important role in regulating heart activities, performance, and morphology including cardiomyocytes growth (7-9). Several studies demonstrated that tissue damage and ischemia followed after chemical and mechanical interactions. In the treatment of these chemical and mechanical interactions is a main cause of protecting myocardium. The lepin hormone with suppressing heart tissue inflammation plays the protective effect role in heart (10, 11). These peptides with kinase activity, its related paths including RIS, PI3K AKT,and P44/42 as well as inhibiting MPTP can cause tissue protection (10). Leptin can control apoptosis factors, prevent cell apoptosis, especially muscle cells, and decrease the risk of infarction. In children with ß-thalassemia, fat cells are not able to synthesize adequate amounts of leptin and therefore, leptin starts to decrease (12,13). Repeated red blood cell transfusion and iron over load in patients with thalassemia lead to chronic hemolysis. Erythrocyte and platelet adhesion to endothelial cells caused reduction of leptin effectiveness. Indeed leptin is produced by epicardium of the heart tissue. HL1 Cells create a classified layer on the fractured parts of fat in a single layer and increase the reduced leptin in these parts and prevent of apoptosis, and play a protective role against death cardiac cells (14). In the other hand, leptin receptor exists in human endothelial cells. Leptin has also considered as a factor in regulating the blood pressure when the endothelial cell is damaged and when atherosclerosis occurs , it provides proper condition to hypertension as a risk factor for heart disease and infarction. (15, 16). Cardiac involvement is the most important complication in patients with thalassemia and the most common cause of death among these patients (17). Troponin t is the selected biomarker for determining heart damage and according to the studies in which done in these matters, increasing of tropinin can be a specific cause in myocardial injury (17, 18). Indeed, troponin t is a structural protein of myocytes which is found in the cytoplasm of cardiac cells (19, 20) and is effective in the contraction of heart cells. Therefore, troponin regulates performance and myosin functions. Releasing of lysosomic enzymes into the cytoplasm caused myocytes damage, cell death, extensive infarction, and release of troponin t in serum (21). 

This study aimed to assess the relationship between levels of leptin and troponin serums in patients with major thalassemia.

## Material and Methods

In this cross-sectional (descriptive- analytical) study, 70 children aged between 2 and 18 years were selected among children who had referred to Ali Ebne Abitaleb Hospital randomly to get a pack red cell for transfusion and, diagnosed by hemoglobin electrophoresis. The participants were not treated with any heart medication and had no kidney disease, diabetes, fever, systemic diseases filling out the consent by parents, those children were enrolled for current study. While fasting 2ml blood was drawn from each sample. Samples were centrifuged with 3000 rpm at 5 ° C for 10 minutes. Separated serums were held in a -20° C until the time of measuring leptin and troponin. Considering the cold chain, serums were transferred to the Biochemistry Laboratory of Zahedan University of Medical Sciences (ZaUMS). Then, 250 microns from serums were used to measure troponin and same process for measuring leptin in which was evaluated using ELISA kit. Data were collected and analyzed with SPSS 20. Descriptive statistics are shown by frequency tables and for inferential statistics, independent t-tests, ANOVA, Pearson correlation, and Chi-square were used. P < 0.05 was considered as significant level.

## Results

In the present study, 70 children were selected among those who had referred to Ali Ebne abitaleb hospital in Zahedan and fulfilled the study criteria. The mean age of participants was 13.75± 5.91, 12.63 ±1.12 for boys and 14.70 ± 0.88 for girls. Table 1 shows the mean of ferretin and Troponin in girls and boys were non significant, when the mean of leptin in girls (2.47 ± 3.13) and boys (0.96 ± 1.08) had a significant difference (t=2.74, p =0.009). However, there was no significant correlation between levels of leptin, troponin, and ferritin serums (p ≥ 0.05) (Table 2). Table 3 showed a significant difference in the means of leptin, ferrittin, and BMI in age groups. In the case of troponin the results showed a non significant difference for age groups.

**Table I:** T-test analysis for variables based on gender groups

**Variable**	**sex**	**Mean±SD**	**t**	**P value**
**leptin**	Girls	2.47±3.13	2.74	0.009
	boys	0.96±1.08		
**Ferretin**	Girls	984.85±362.05	1.006	0.318
	boys	899.05±347.31		
**Troponin**	Girls	20.92±37.5	1.1	0.275
	boys	13.29±9.94		

**Table II:** Matrix correlation for variables in study

**Variables**	**Statistics**	**Leptin**	**ferretin**	**troponin**	**age**
**BMI**	Pearson C.	0.374	0.085	-0.043	0.226
	Sig. level	0.002	0.487	0.727	0.060
**Leptin**	Pearson C.		-0.078	-0.114	0.248
	Sig. level		0.528	0.357	0.041
**ferretin**	Pearson C.			0.018	0.607
	Sig. level			0.884	0.000
**troponin**	Pearson C.				-0.029
	Sig. level				0.813

**Table III:** Results of ANOVA test for variables based on Age groups with following test of tukkey

**Dependent** **Variable**	**(I)**	**Mean**	**(J)**	**i-j**	**p**	**95% C I**		**F**	**P**
						LB	UB		
**BMI**	<5 ys	17.41±2.39	(5 , 10) ys	2.84	NS	-0.76	6.44	6.156	0.004
			>=10 ys	0.10	NS	-3.44	3.63		
	(5 , 10) ys	15.1±1.89	<5 ys	-2.84	NS	-6.44	0.76		
			>=10 ys	-2.74	<0.05	-4.00	-1.48		
	>=10 ys	16.88±2.37	<5 ys	-0.10	NS	-3.63	3.44		
			(5 , 10) ys	2.74	<0.05	1.48	4.00		
**leptin**	<5 ys	0.37±0.22	(5 , 10) ys	-0.02	NS	-0.47	0.43	4.049	0.022
			>=10 ys	-1.80	<0.05	-2.78	-0.82		
	(5 , 10) ys	0.55±0.44	<5 ys	0.02	NS	-0.43	0.47		
			>=10 ys	-1.78	<0.05	-2.78	-0.78		
	>=10 ys	2.32±2.83	<5 ys	1.80	<0.05	0.82	2.78		
			(5 , 10) ys	1.78	<0.05	0.78	2.78		
**ferretin**	<5 ys	670.22±33.79	(5 , 10) ys	-68.65	NS	-564.67	427.37	6.6	0.002
			>=10 ys	-338.49	NS	-817.62	140.64		
	(5 , 10) ys	769.08±212.37	<5 ys	68.65	NS	-427.37	564.67		
			>=10 ys	-269.84	<0.05	-481.03	-58.64		
	>=10 ys	1040.96±358.08	<5 ys	338.49	NS	-140.64	817.62		
			(5 , 10) ys	269.84	<0.05	58.64	481.03		
**troponin**	<5 ys	14.47±9.05	(5 , 10) ys	-13.96	NS	-61.11	33.18	1.337	NS
			>=10 ys	-3.44	NS	-14.63	7.74		
	(5 , 10) ys	28.15±58.63	<5 ys	13.96	NS	-33.18	61.11		
			>=10 ys	10.52	NS	-35.70	56.74		
	>=10 ys	14.54±10.60	<5 ys	3.44	NS	-7.74	14.63		
			(5 , 10) ys	-10.52	NS	-56.74	35.70		

## Discussion

This study aimed to assess the relationship between levels of leptin and troponin serums in patients with major Beta thalassemia. The analysis showed a significant difference in the mean of leptin in girls and boys had but not for ferritin and troponin. Significant correlation between troponin and ferretine was not observed whereas leptin depicted a positive relationship with age. In a study by Smith CC on the role of adipose- kinesis in pathophysiology of the cardiovascular heart disease, the protective role of leptin was proposed (10). Ku IA, et al investigated the relationship between leptin and cardiovascular events in patients with coronary disease and concluded that leptin reduction can be considered as a factor which increases the risk of cardiovascular events and death in patients (11). In another study by Yan GT which explored the correlation between leptin and troponin t, concluded that the level of leptin serum and troponin in patients with acute myocardial infarction and coronary atherosclerosis none significantly increased when this increase not significant. While we did a study on patients with major beta thalassemia with the conclusion of being similar results (12). Wang YZ and the colleagues operated a study on mice which proposed that the increase of leptin may affect the growth and development of muscles in contrary our study on human kind (13). Leptin increases inhibition of protein degradation and production of muscle cells, also leptin suppressed heart muscle inflammation and this is useful in the protection and treatment of heart disease (10, 15). Our results showed that leptin serum level was significantly higher for ages older than 10 and this issued fact introducing leptin as a biomarker for evaluating cardiac involvement in patients with major beta thalassemia. A study by shahramian revealed that Troponin is a selected marker for patients, who suffer from heart disease, and released from myocytes and is spilled into the serum and is measured in case of micro infarct, he also resulted that troponin level had significant positive correlation with age. This is in contrast with the present study (3). Since after age eight in patients with thalassemia cardiac involvement occurs due to repeated red blood cell transfusion. The main reason of frequent transfusion in patients with thalassemia is ineffective and increasing erythropoiesis of bone marrow, which leads to an increase in plasma volume and high cardiac output, and finally creates cardiomyopathy. In these cases, cardiac involvement is created without any infarction (19, 20, and 22). Our results related to the association of leptin with age were consistent with mentioned studies. Iron is deposited on the Hiss bundle and Purkinje cell and when it reaches to the critical surface, dilated cardiomyopathy occurs which is the most common cause of heart disease in these patients. Moreover, during the second and third decades of their lives, these patients will face with iron deposition in parenchymal myocardial tissues that leads to arrhythmias, systolic, diastolic dysfunctions and CHF (21, 23). Our results showed that ferretin was higher for ages older than 10 years in which shows iron overload in body systems such as, cardiovascular system. Perrone fin dines showed that in thalassemia patients, adipose tissue is not able to guarantee adequate leptin production just when the highest leptin secretion is needed and Perrone suggested that this inadequate leptin secretion may be a multi cause of the unbalanced in pubertal timing. These findings are in line with the results of our study that proved the serum leptin level had highest amount for the ages older than 10 years (24).Musallam KM did a 10 years follow-up study on serum ferritin level in transfusion-independent patients with beta-thalassemia and concluded that the ferritin level increased after ten years for the same patients which is in line with our findings (25).

## Conclusion

Our results showed that leptin increased with age and since from the age of 10, the cardiac involvement begins to increase, we then can conclude that there is a remarkable and positive association between leptin and cardiac involvement. Based on Noori, it can be concluded that leptin’s change is independent from troponin, which is related to the cardiac involvement and the age of cardiac involvement in patients with beta-thalassemia major is 10–15 years. According to the results of the present study, leptin may be used in children with major thalassemia as a selected Cardiac marker involvement screening. 
